# Pulmonary Manifestations in Patients With Hematologic Malignancies: In Pursuit of an Accurate Diagnosis

**DOI:** 10.7759/cureus.77418

**Published:** 2025-01-14

**Authors:** Jose C Alvarez-Payares, Daniel Andres Ribero Vargas, E. U. Suárez, Daniel Barrera-Correa, Juan David Vélez Aguirre, Juan C Hernandez-Rodriguez, Sara I Ramirez-Urrea

**Affiliations:** 1 Hematology, University of Antioquia, Medellín, COL; 2 Internal Medicine, University of Antioquia, Medellín, COL; 3 Hematology, Fundación Jiménez Díaz University Hospital, Madrid, ESP; 4 Vascular Medicine, University of Antioquia, Medellín, COL; 5 General Medicine, University of Antioquia, Medellín, COL; 6 Pediatric Medicine, Pontifical Bolivarian University, Medellín, COL

**Keywords:** complications, diagnostic imaging, differential diagnosis, hematologic neoplasms, mortality, pulmonary disease

## Abstract

Pulmonary involvement is common in patients with hematologic malignancies (HMs) and varies depending on the underlying condition, including lymphoproliferative disorders, acute leukemia, myelodysplastic syndrome, and allogeneic stem cell transplantation. Pulmonary complications are a frequent cause of morbidity and mortality in these patients, often resulting from the immunosuppressive effects of the disease or its treatment. The clinical manifestations of these complications are nonspecific, and their differential diagnosis is broad, encompassing both infectious and noninfectious causes. A thorough clinical assessment requires consideration of factors such as the patient's history, baseline immune status, treatment regimens, time since the last chemotherapy, and environmental exposures. Radiographic imaging, particularly high-resolution CT, plays a critical role in evaluating these complications, helping clinicians identify distinct patterns of pulmonary involvement. Therefore, a personalized diagnostic approach is essential, and multidisciplinary management is crucial for optimal patient care.

## Introduction and background

The global burden of cancer continues to rise annually. The US CDC predicts an increase in diagnoses, projecting up to 19 million cases by 2025, up from 14 million in 2012 [1]. This trend is also observed in hematologic malignancies (HMs). In the most recent study on disease burden, the incidence of leukemias and non-Hodgkin lymphomas (NHLs) showed significant increases of 26% and 46%, respectively, between 2006 and 2016 [2].

HMs encompass a diverse group of clonal diseases, characterized by abnormal proliferation and impaired apoptosis in various blood cell lines. These include myeloid, lymphoid, and plasma cell disorders [3].

Pulmonary complications in these diseases are common causes of both morbidity and mortality. For example, approximately 50% of patients with an HM will experience a pulmonary infection during treatment [1]. In fact, 85% of individuals with Hodgkin lymphoma (HL) and 66% of those with NHL may present with pulmonary findings [3]. These complications are often linked to the systemic immunosuppressive effects of the disease itself (e.g., neutropenia in acute leukemia, hypogammaglobulinemia in multiple myeloma (MM)), antineoplastic therapies (including cell therapy and immune checkpoint inhibitors (ICIs)), or stem cell transplantation [4]. Furthermore, the extensive alveolar and vascular surface in the lungs, coupled with high cellular turnover, makes these patients particularly susceptible to the adverse effects of cytotoxic treatments [1]. As a result, the differential diagnosis of pulmonary manifestations in patients with HMs is broad, encompassing both infectious and noninfectious causes [3].

This review aims to explore the various pulmonary complications that may arise in patients with HMs, with the goal of establishing an accurate differential diagnosis.

## Review

Diagnostic approach

When a patient with any HM presents with pulmonary manifestations, the first step is to assess individual risk factors. This involves obtaining detailed information regarding the timeline, patient characteristics, baseline immune status, treatment regimens, the timing of the most recent therapy cycle, and any potential environmental exposures.

With this information, the physician can begin to narrow down the most likely causes of the clinical presentation. Essential diagnostic tools, such as chest CT imaging and microbiological studies from bronchoalveolar lavage (BAL), will aid in this process. Pulmonary complications will then be categorized into infectious and noninfectious causes, taking into account the specific HM involved.

Lymphomas

**Figure 1:**
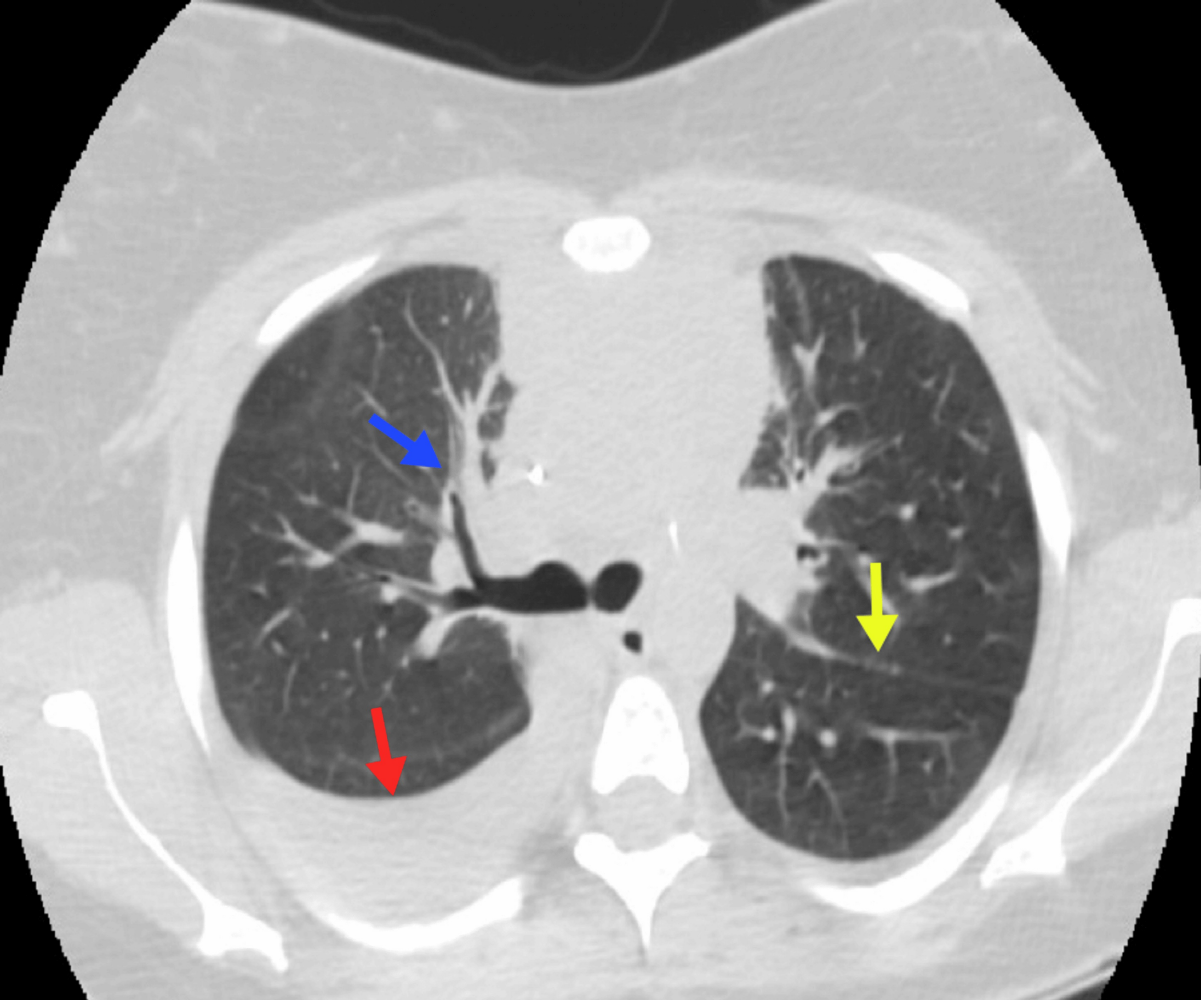
Primary mediastinal NHL A 24-year-old female patient with primary mediastinal large B-cell lymphoma. A heterogeneous mass is observed in the anterior mediastinum (blue arrow), extending to the medial mediastinum. Bilateral pleural effusion with right predominance is noted (red arrow), along with lingular atelectasis (yellow arrow). NHL, non-Hodgkin lymphoma Image courtesy: Pulmonology Service of the Internal Medicine Department, University of Antioquia. Consent for open access publication was obtained from the patient.

Pulmonary involvement is most common in lymphomas when there is generalized or extra-thoracic disease [5]. Parenchymal lesions are found in 24% of NHLs and up to 85% of HL. In HL, pulmonary involvement is often associated with intrathoracic lymphadenopathy, whereas NHL rarely involves the mediastinum, except in cases such as primary mediastinal lymphoma (Figure 1) and gray zone lymphoma [6]. Primary pulmonary lymphomas are rare, accounting for only 0.5% of all pulmonary neoplasms [5].

The following tomographic patterns have been described:

Nodular

**Figure 2:**
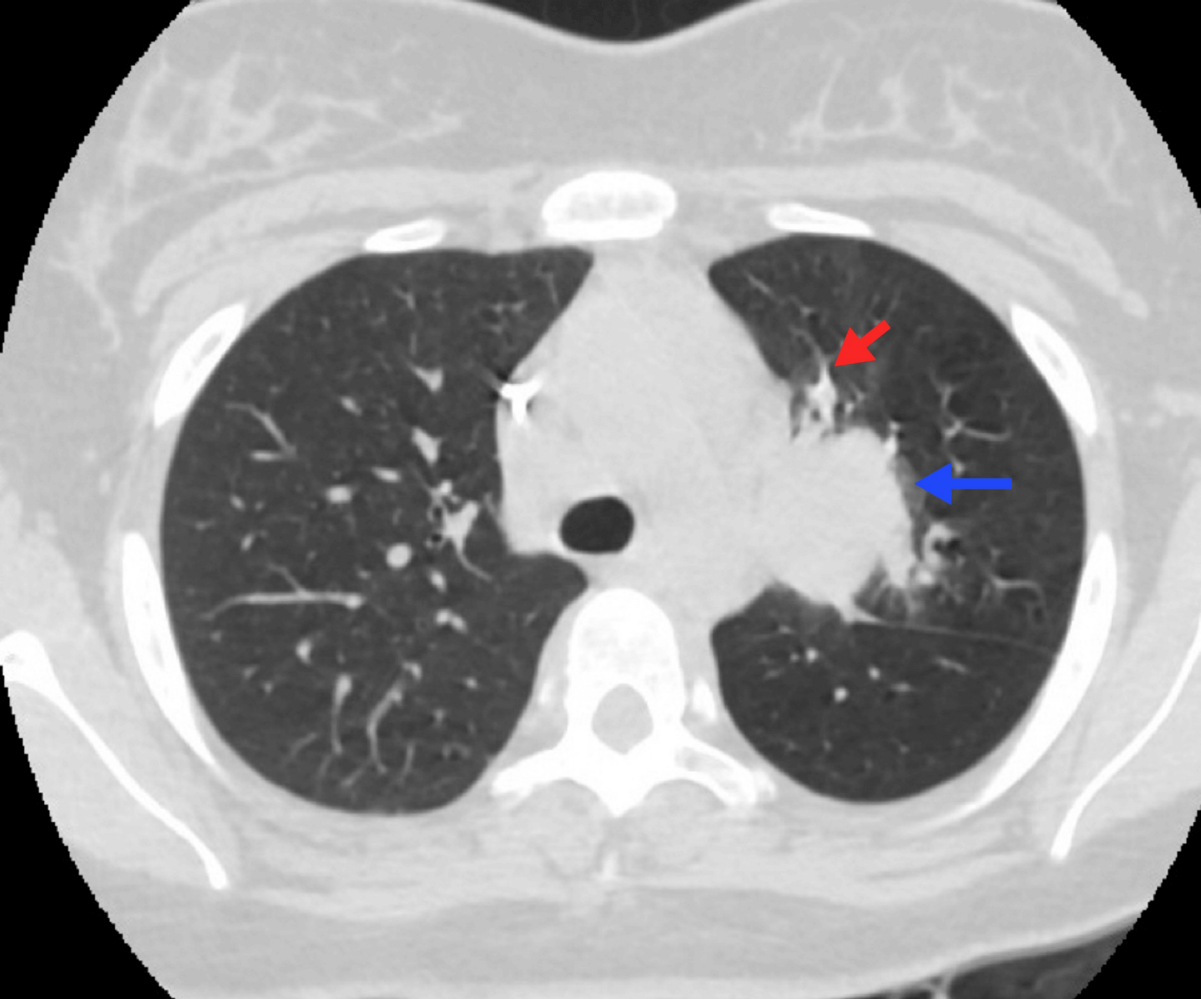
Adenopathies and pulmonary nodules An 18-year-old patient with HL, nodular sclerosis variant. A lymph node conglomerate is observed toward the aortopulmonary window (blue arrow), with various nodular opacities in the lung parenchyma due to invasion by proximity (red arrow). HL, Hodgkin lymphoma Image courtesy: Pulmonology Service of the Internal Medicine Department, University of Antioquia. Consent for open access publication was obtained from the patient.

In 86% of cases, multiple nodules are present, with up to 66% being bilateral. Some nodules may have irregular or ill-defined margins, or even cavitation, particularly in high-replication tumors such as large B-cell diffuse lymphomas (Figure 2) [7].

Consolidations

**Figure 3:**
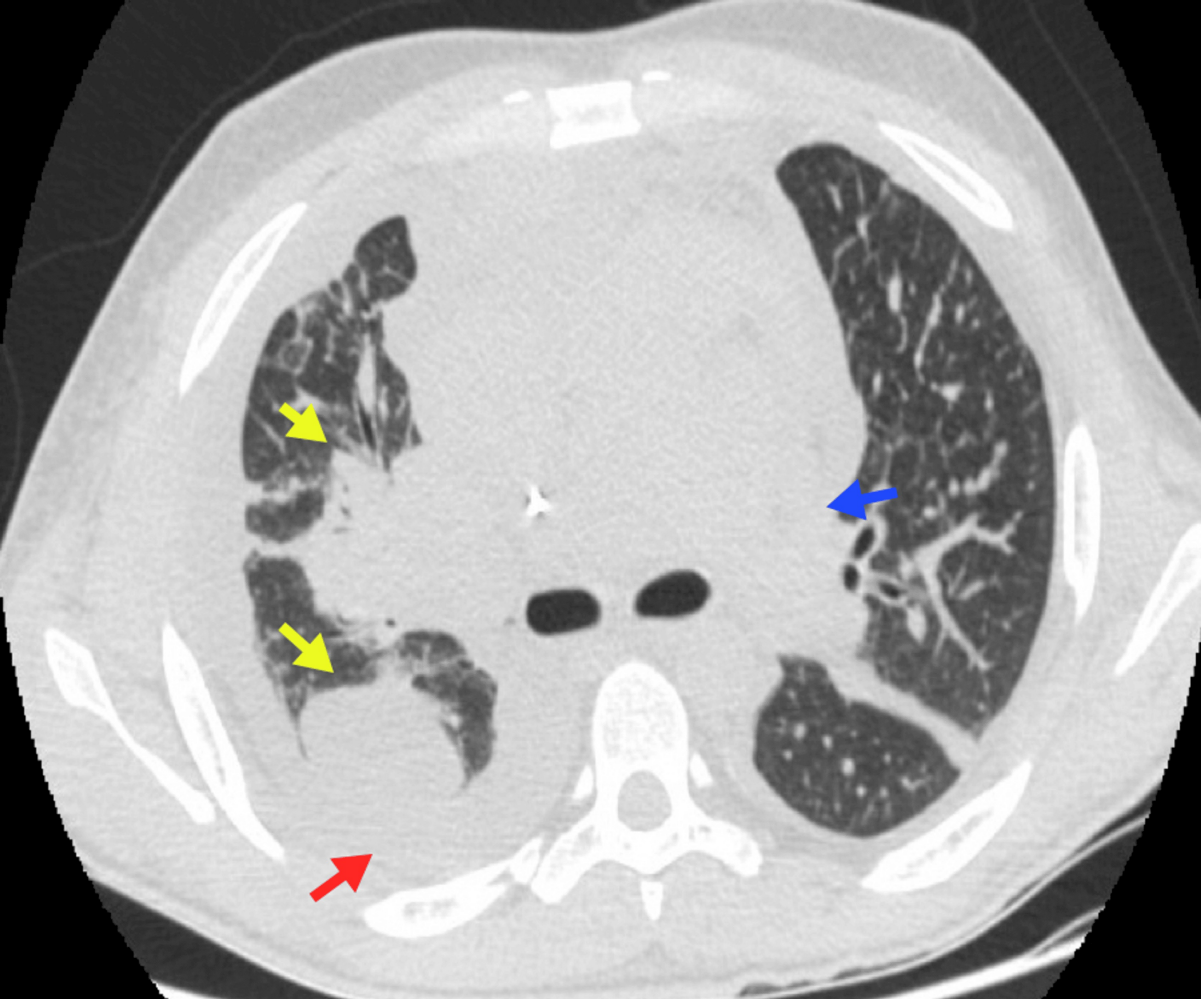
Consolidations A 30-year-old male patient with classic HL and relapses of high-grade B-cell lymphoma, NOS. Neoplastic involvement of the mediastinum is observed (blue arrow), along with bilateral pleural effusion (red arrow) and right lung consolidations (yellow arrows). HL, Hodgkin lymphoma; NOS, not otherwise specified Image courtesy: Pulmonology Service of the Internal Medicine Department, University of Antioquia. Consent for open access publication was obtained from the patient.

This finding can range from small, localized areas to complete lobar involvement or, less commonly, multifocal or multilobar involvement, which is typically indolent and grows slowly [8]. The “angiogram” sign has been described, characterized by the enhancement of a pulmonary vessel within a consolidation zone. Additionally, a perilesional ground-glass opacity has been observed in some cases of MALT lymphoma, although its diagnostic value remains uncertain (Figure 3) [6].

Lymphangitis/Perilymphangitis

**Figure 4:**
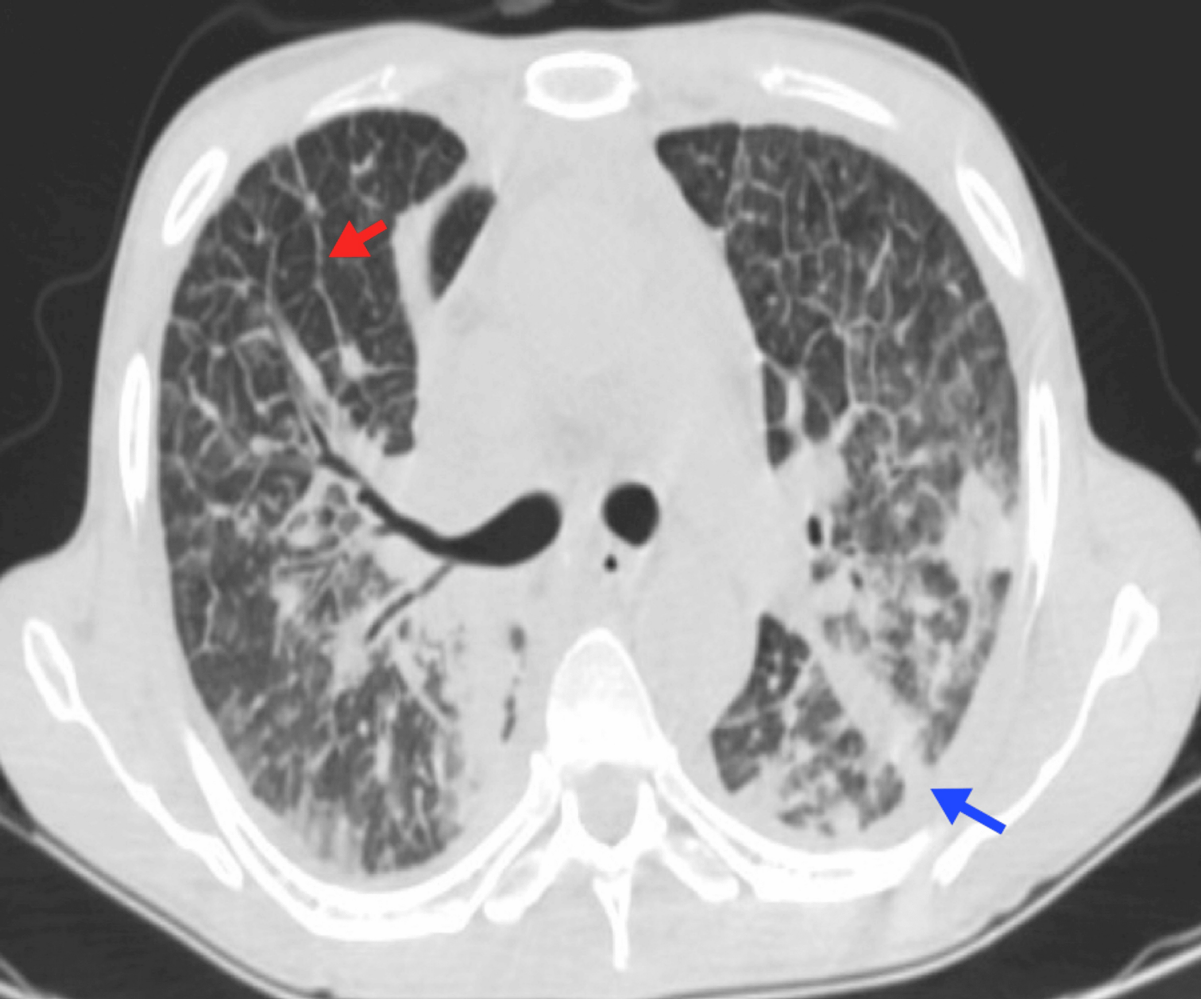
Lymphangitic carcinomatosis A 43-year-old patient with a medical history of common variable immunodeficiency and HL. Mild pleural and pericardial effusion is observed (blue arrow), along with widespread interlobular septal thickening suggesting lymphangitic carcinomatosis (red arrow). HL, Hodgkin lymphoma Image courtesy: Pulmonology Service of the Internal Medicine Department, University of Antioquia. Consent for open access publication was obtained from the patient.

Lymphomas with secondary pulmonary involvement often present with a bronchovascular pattern resembling lymphangitis, characterized by bronchovascular loops and interlobular septal thickening in up to 41% of cases (Figure 4) [9].

Ground Glass

This is the least common pattern, with only a limited number of case reports.

Leukemias

**Figure 5:**
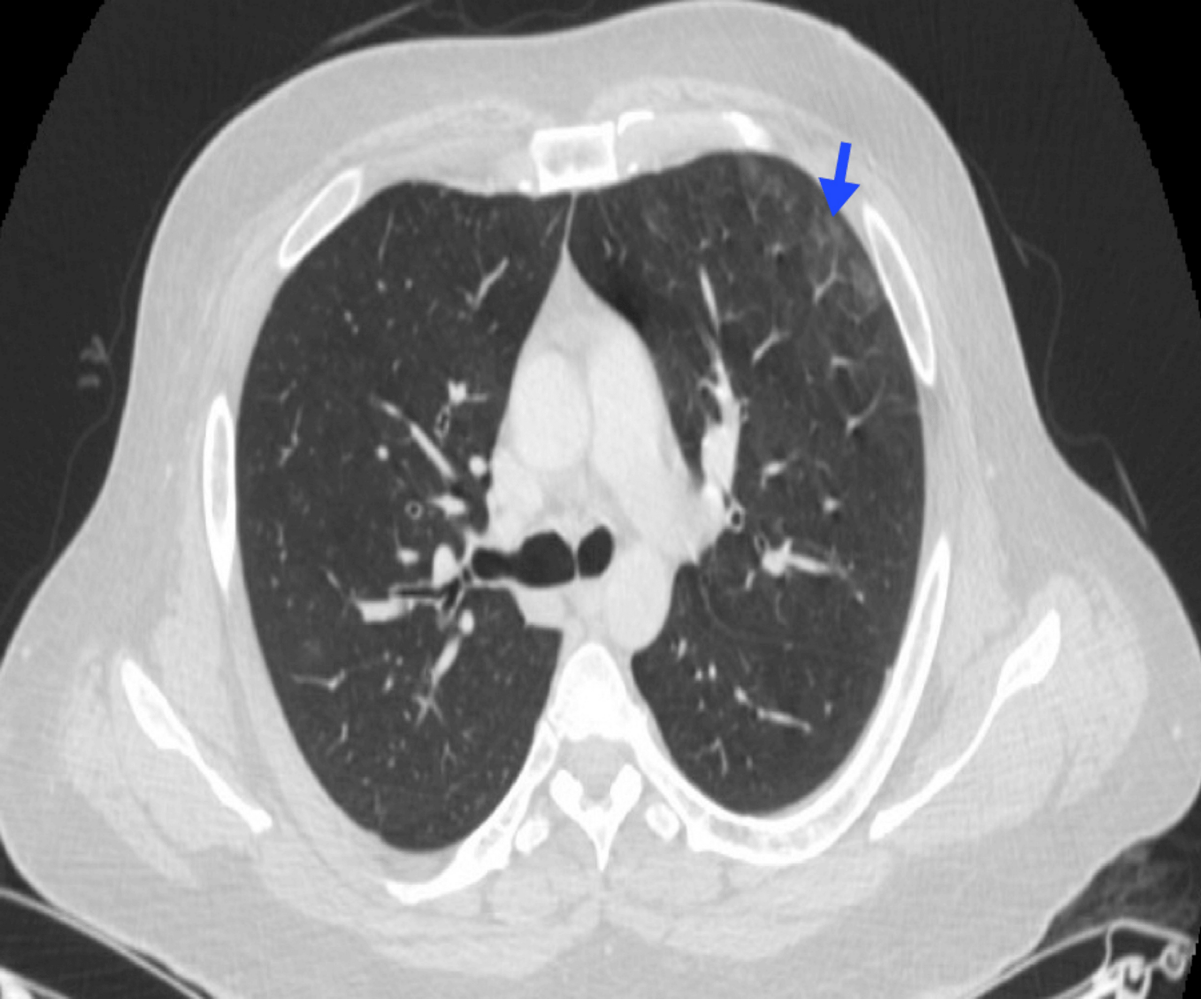
Leukemia lung involvement A 27-year-old male patient with the common phenotype of ALL. Chest CT reveals peripheral ground-glass opacities in the upper left lobe, along with central acinar micronodules of random distribution (blue arrow). Infectious causes were ruled out through BAL. ALL, acute lymphoblastic leukemia; BAL, bronchoalveolar lavage Image courtesy: Pulmonology Service of the Internal Medicine Department, University of Antioquia. Consent for open access publication was obtained from the patient.

Lymphoid lineage leukemias, such as acute lymphoblastic leukemia (ALL) and chronic lymphocytic leukemia (CLL), may exhibit similar pulmonary involvement to that seen in lymphomas (Figure 5).

**Figure 6:**
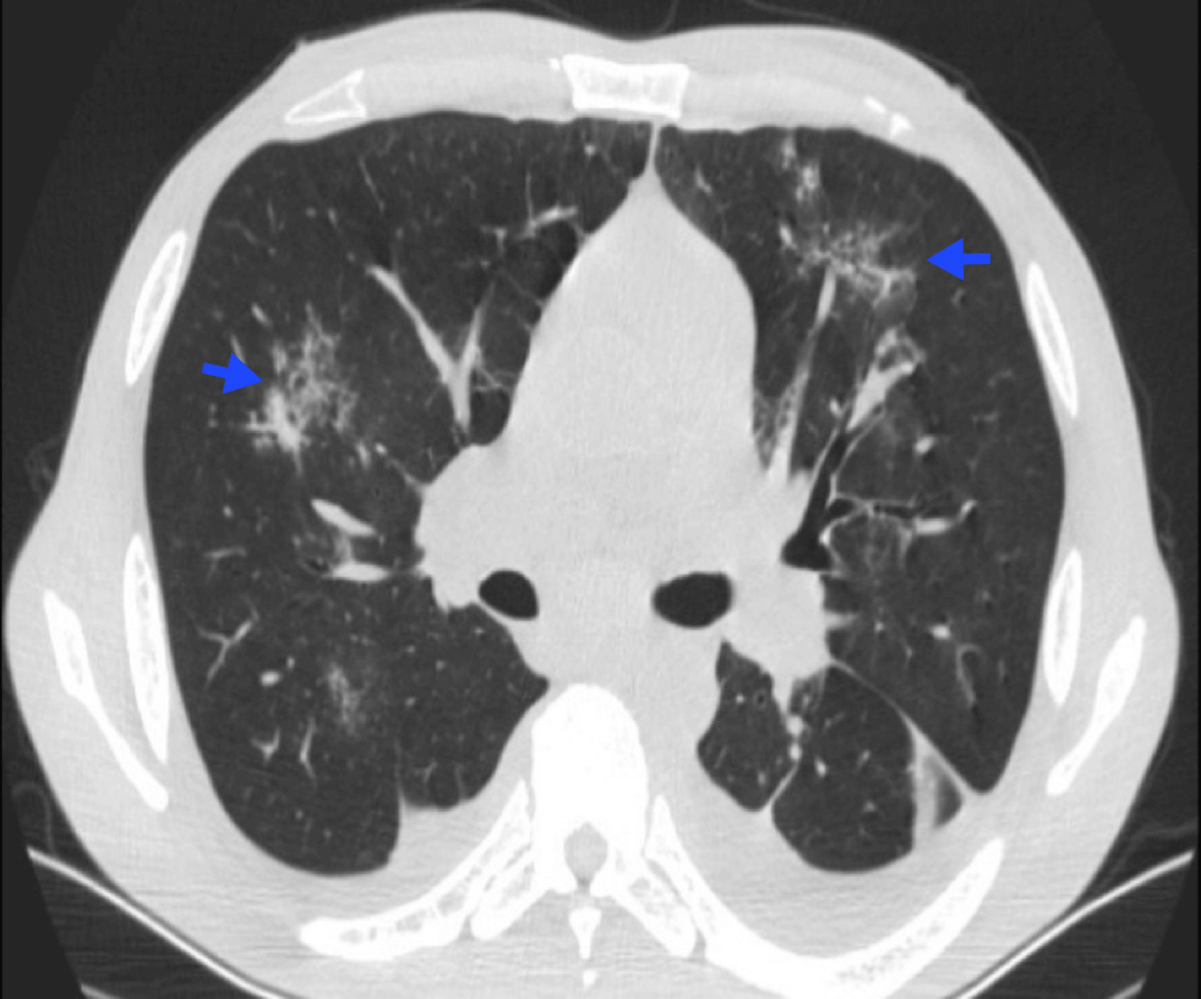
Pulmonary involvement in CLL A 55-year-old male patient with a medical history of CLL. Bilateral peribronchovascular ground-glass opacities are observed (blue arrows). Pulmonary cytology from BAL was positive for lymphoid infiltration. BAL, bronchoalveolar lavage; CLL, chronic lymphocytic leukemia Image courtesy: Pulmonology Service of the Internal Medicine Department, University of Antioquia. Consent for open access publication was obtained from the patient.

In patients with CLL, up to 9% may exhibit pulmonary involvement (Figure 6).

Peribronchial or perivascular involvement is the most common pattern, followed by interstitial, nodular, and centrilobular patterns [10]. Hilar and mediastinal involvement have also been reported [11]. Pleural effusion has been observed in CLL, with frequencies ranging from 3% to 16% in cadaveric studies [11]. The pleural fluid is typically exudative with lymphocytic predominance, although hemorrhagic or chylous fluid has also been described [12]. Additionally, malignant pleural effusion occurs in 7% of cases, mostly in advanced disease or Richter syndrome [10].

Myeloid lineage leukemias are also associated with pulmonary involvement. Mediastinal involvement has been documented in cadaveric studies of patients with a history of acute myeloid leukemia (AML) [13]. Myeloid sarcoma (MS), a rare extramedullary malignancy of immature cells, commonly involves the mediastinum [14]. MS usually presents before or during AML diagnosis but can also occur in myelodysplastic syndrome (MDS) and other myeloproliferative neoplasms (MPNs). Imaging of MS typically reveals nodular infiltrates, interstitial infiltrates, and consolidation [15].

AML can involve the tracheobronchial tree as a bronchial chloroma, a rare mass composed of myeloblasts. Pleural effusions are infrequent in MPNs [16].

**Figure 7:**
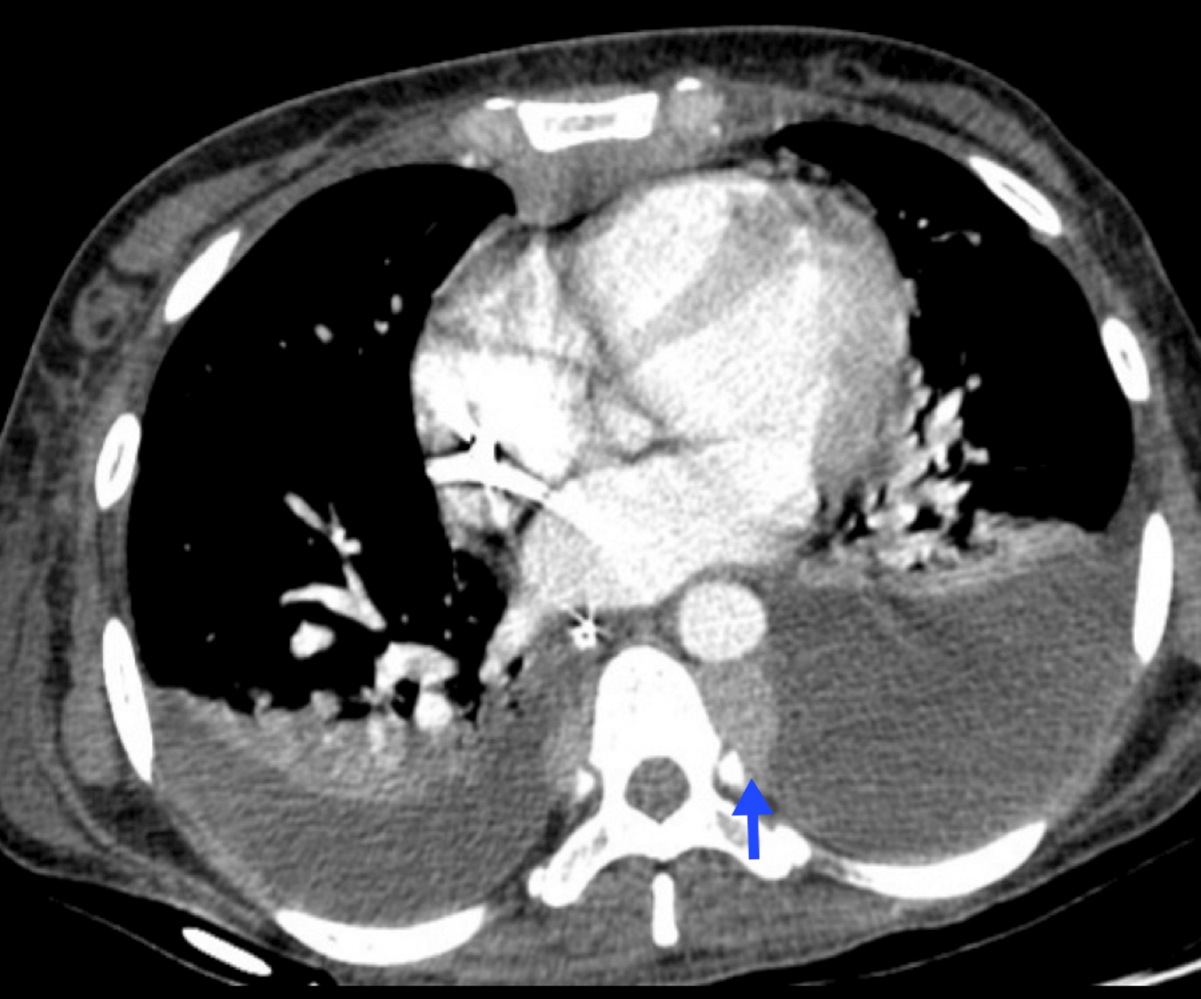
EMH A 28-year-old female patient with IgG lambda MM. Paravertebral and left-sided soft tissue thickening (blue arrow), a finding associated with EMH. EMH, extramedullary hematopoiesis; MM, multiple myeloma Image courtesy: Pulmonology Service of the Internal Medicine Department, University of Antioquia. Consent for open access publication was obtained from the patient.

Extramedullary hematopoiesis (EMH) is another phenomenon seen in chronic myeloid leukemia (CML), other MPNs, and plasma cell dyscrasias (Figure 7).

Pulmonary involvement due to EMH can manifest as pleural effusion, subglottic stenosis, pulmonary hypertension, or even acute respiratory insufficiency [17].

MPNs and MDSs

Pulmonary involvement in MPNs is diverse, although few registries have been published [18]. One such manifestation is EMH, which is associated with medullary failure due to myelofibrosis [19].

While EMH has primarily been described in the spleen, liver, and lymph nodes, it can occur in any organ. Thoracic involvement is rare, and most cases present as tumors in the posterior-inferior mediastinum, often in paraspinal positions. These tumors typically appear as asymmetric, nonpulsatile, noncalcified paravertebral masses with benign behavior, and they may be unilateral or bilateral. Some cases may be mistaken for neurogenic tumors [20].

Chloromas have been reported in patients with polycythemia vera (PV), myelofibrosis, and hypereosinophilic syndromes, with an overall frequency of up to 39% in MPNs [21]. While intrathoracic chloromas are rare, around 50 cases have been reported. These tumors generally present as prominent mediastinal masses on chest X-rays and may involve the pleura, lungs, pericardium, and hilum. Chloromas are associated with bronchial compression and a poor prognosis [22].

Additionally, MDSs have been linked to autoimmune paraneoplastic phenomena, such as Raynaud’s syndrome, Sjögren’s syndrome, systemic lupus erythematosus, and polychondritis. As a result, MDS may present with a variety of lung involvements related to these diseases [23]. Pulmonary involvement in these cases is typically associated with systemic involvement and often presents as diffuse alveolar infiltrates, with up to 50% of patients developing severe acute respiratory failure [18].

Sweet’s syndrome, an autoinflammatory phenomenon, occurs in up to 9% of MDS cases and 7% of patients with chronic-phase CML. Pulmonary involvement in Sweet’s syndrome is characterized by multiple diffuse alveolar infiltrates, indicating neutrophil migration to the lung parenchyma [24].

Besides neutrophilic infiltrates, other lung manifestations have been described in MPN/MDS with eosinophilia. The most common findings include diffuse pulmonary infiltrates, unilateral alveolar opacities, and bilateral pleural effusions [18].

Plasma cell neoplasms

In MM, up to 10% of patients may experience pulmonary involvement during the course of the disease. The most common manifestations include bacterial and fungal infections, pleural effusion, pleural and parenchymal plasmacytomas, and, less frequently, diffuse pulmonary involvement [25]. Primary pulmonary plasmacytomas are rare, with only a few case reports in the literature [26].

Unicentric Castleman’s disease, a lymphoproliferative neoplasm characterized by plasma cell hyperplasia, typically presents with multiple lymphadenopathies in various anatomical regions, although most cases involve mediastinal involvement [27].

In primary amyloidosis, the systemic variant can present with pulmonary involvement in up to 50% of patients, although radiographic findings are less frequent. Three types of presentations have been described: (a) diffuse interstitial deposits; (b) solitary or multiple nodules; and (c) submucosal tracheobronchial deposits, with the latter being the most common [28].

High-resolution CT (HRCT) has revealed several radiographic patterns, including tracheal stenosis, bronchial wall thickening, calcified amyloid deposits, and multiple nodules. In the diffuse form, nonspecific widespread opacities (both alveolar and interstitial) are noted, along with subpleural nodules containing calcified zones [29].

The most common complications associated with HMs are summarized below. Table 1 highlights the major radiographic patterns and their related differential diagnoses.

**Table 1:** Main radiographic patterns and differential diagnoses AML, acute myeloid leukemia; ARDS, acute respiratory distress syndrome; CLL, chronic lymphoid leukemia; CML, chronic myeloid leukemia; CMV, cytomegalovirus; EMH, extramedullary hematopoiesis; HHV-6, human herpes virus 6; MDS, myelodysplastic syndrome; MM, multiple myeloma; MPN, myeloproliferative neoplasm; MS, myeloid sarcoma; PAP, pulmonary alveolar proteinosis; PV, polycythemia vera; PVOD, pulmonary veno-occlusive disease; TKI, tyrosine kinase inhibitor; TRALI, transfusion-related acute lung injury

Radiographic pattern or finding	Etiologies	Radiographic pattern or finding	Etiologies
Nodules	Acute: infection (necrotizing bacteria such as *Pseudomonas*, *Staphylococcus aureus*, *Klebsiella *spp., and *Aspergillus *spp. infections)	Pulmonary hypertension	MPN
	Subacute/chronic: infection (fungi, *Nocardia*, and Mycobacteria), organizing pneumonia, lymphoma, lymphoid leukemias, and primary pulmonary amyloidosis		TKIs (mainly dasatinib)
Masses	EMH (CML and MPN)	Alveolar occupation	Acute: infection (mainly bacterial)
	MS (AMS, MDS, and MPN)		
	Chloromas (PV, myelofibrosis, hypereosinophilic syndromes, and AML)		Subacute/chronic: infection (fungi, *Nocardia*, *Actinomyces* spp., and mycobacteria), drug-induced toxicity, organizing pneumonia, Sweet’s syndrome, and lymphoma
	Pulmonary plasmacytoma		
Interstitial infiltrates	Acute: infection (late* Pneumocystis jirovecii* infection, viruses, and atypical bacteria), pulmonary edema, ARDS, and TRALI	Ground glass	Acute: infection (*P. jirovecii *infection, CMV, HHV-6, respiratory viruses, and atypical bacteria), alveolar hemorrhage, acute radiation pneumonitis, eosinophilic pneumonia, pulmonary edema, ARDS, and TRALI
	Subacute/chronic: EMH, autoimmune paraneoplastic syndromes, hypereosinophilic syndrome, PAP, organizing pneumonia, lymphoid leukemias, and primary pulmonary amyloidosis		Subacute/chronic: infection (CMV and atypical bacteria), drug- or radiation-induced toxicity, PAP, PVOD, and lymphomas

Noninfectious pulmonary complications

Pulmonary Hemorrhage

Pulmonary hemorrhage is the most common noninfectious pulmonary complication in acute leukemias [30]. Diffuse alveolar hemorrhage may occur in up to 20% of hematopoietic stem cell transplantation (HSCT) recipients, typically within the first few weeks after transplantation. It is also associated with thrombocytopenia and infectious diseases [31].

Symptoms are generally nonspecific and acute, particularly when related to HSCT. These symptoms include sudden dyspnea, nonproductive cough, fever, hypoxemia, and anemia. Hemoptysis is rare, and symptoms may be mild despite the rapid progression of imaging findings [32].

Chest X-ray may reveal a ground-glass pattern with patchy consolidations, while HRCT may show ground-glass opacities, consolidations, a reticular pattern, and a crazy paving pattern [33].

A definitive diagnosis is made through BAL, which reveals an elevated blood content (hemosiderin-laden macrophages >20%), provided there is no evidence of infection [34].

Pulmonary Leukostasis

Pulmonary leukostasis is a common complication in patients with hyperleukocytosis (leukocyte count: >100,000 cells per milliliter), particularly in AML. The excessive number of cells accumulates in the small-caliber blood vessels of various organs, including the lungs, heart, brain, and testicles, leading to diverse symptoms. Some studies have identified leukemic pulmonary infiltration as the second most common pulmonary complication in patients who have not undergone HSCT [35].

The clinical presentation is nonspecific, with fever, cough, and dyspnea being the most common symptoms. Alveolar or interstitial opacities may be visible on chest X-rays, although imaging may be entirely normal even in patients experiencing severe respiratory insufficiency [36].

**Figure 8:**
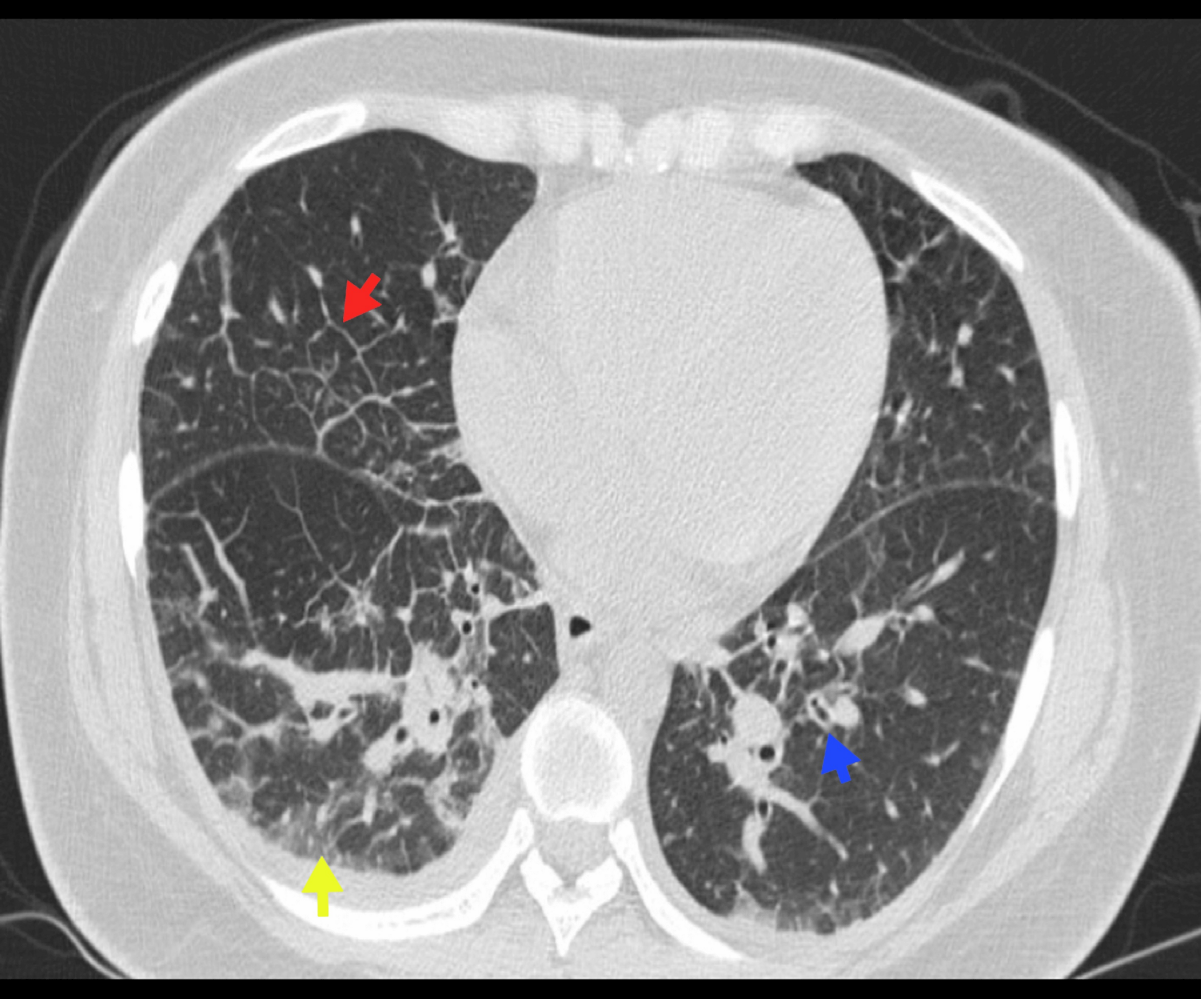
Pulmonary leukostasis A 39-year-old female patient with acute B-cell lymphoblastic leukemia and hyperleukocytosis. Chest CT reveals thickening of the bronchial walls (blue arrow), thickening of the interlobular septa (red arrow), and subpleural ground-glass opacities (yellow arrow). Image courtesy: Pulmonology Service of the Internal Medicine Department, University of Antioquia. Consent for open access publication was obtained from the patient.

The main findings on HRCT (Figure 8) include thickening of bronchovascular loops and prominence of peripheral pulmonary arteries, both of which are associated with areas of leukemic infiltration.

A ground-glass pattern may also be observed, characterized by a nonlobar, nonsegmental distribution, which results from leukemic infiltration of the alveolar space and septum. Hemorrhage and edema are commonly present due to diffuse alveolar damage (DAD) [37]. Although these findings are nonspecific, leukemic pulmonary infiltrates should be considered in any patient with interstitial thickening and a history of leukemia, particularly when hyperleukocytosis is present [3].

Pulmonary Embolism

The association between thromboembolic disease and neoplasms is well established. Studies indicate a seven-fold increase in the risk of venous thromboembolism in patients with any malignancy and a 28-fold increase in those with HM. This risk is particularly elevated during the first few months after diagnosis but tends to decline over time [38].

The risk of cancer diagnosis remains heightened for up to two years after the first nonprovoked event of venous thromboembolism, with a particularly high significance for subsequent diagnoses of ovarian cancer, HL, and NHL [39].

Thrombocytopenia and a disease duration of 12 months are key risk factors for venous thromboembolism, with frequencies ranging from 15% in ALL patients to 8% in AML patients [40].

Additional risk factors identified in studies, mainly involving patients with leukemia and lymphoma, include central venous catheter use, hematopoietic growth factor therapy, high glucocorticoid doses, L-asparaginase therapy, and the use of newer immunomodulatory drugs [16].

**Figure 9:**
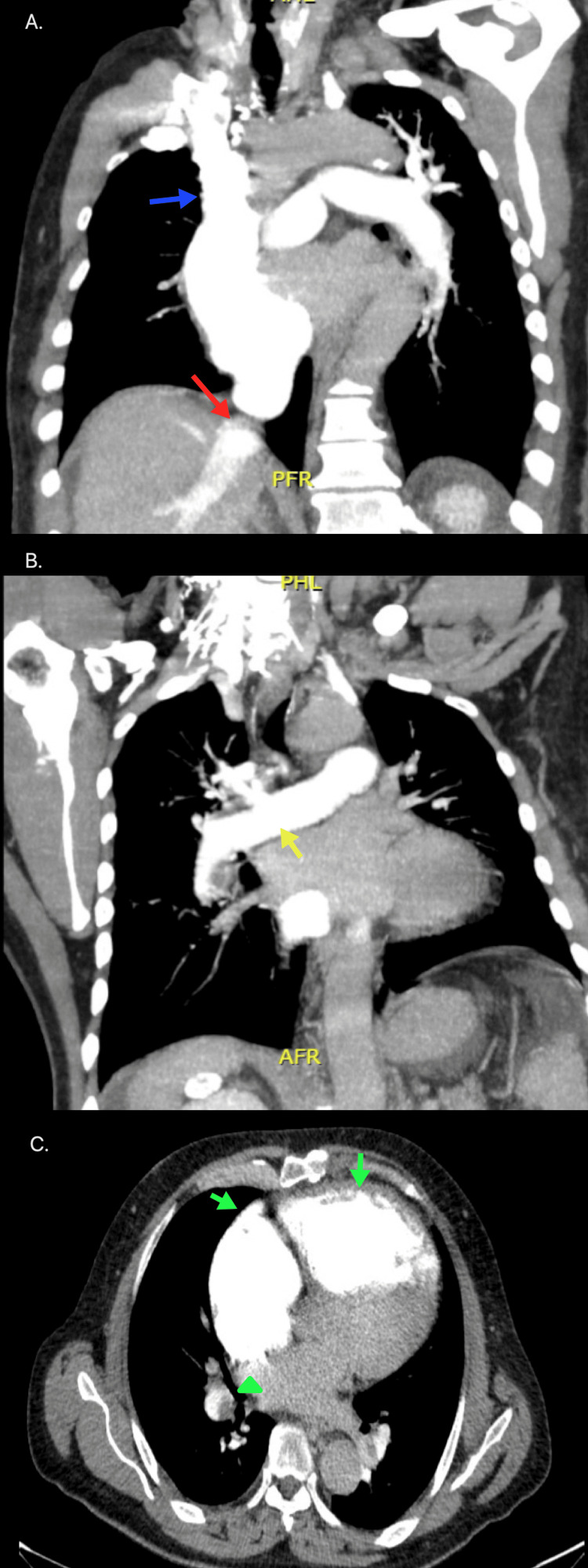
Pulmonary embolism A 60-year-old male patient with severe pulmonary hypertension and JAK2-positive PV. The following findings were observed in chest CT angiography: (A) Dilatation of the superior vena cava (blue arrow) with contrast reflux into the inferior vena cava and opacification of the hepatic veins, indicative of right-sided heart failure (red arrow). (B) Dilation of the pulmonary artery (yellow arrow). (C) Prominence of right-sided cavities (green arrow), with eccentric filling defects involving lobar and segmental arteries, suggestive of chronic pulmonary embolism (green arrowhead). PV, polycythemia vera Image courtesy: Pulmonology Service of the Internal Medicine Department, University of Antioquia. Consent for open access publication was obtained from the patient.

Thromboembolic disease is a significant cause of morbidity and mortality in MPNs, particularly in Philadelphia chromosome-negative conditions like PV and essential thrombocytosis (Figure 9) [41].

The main risk factors in these neoplasms are the underlying cellular anomalies and the pro-inflammatory state associated with the disease. The spectrum of thrombotic involvement ranges from microcirculatory disturbances to venous/arterial thrombosis and chronic thromboembolic disease [42].

Pulmonary Edema

Pulmonary edema is common during the administration of chemotherapeutic agents and other intravenous infusions or as an early complication of bone marrow transplantation [3]. Its etiology is most likely multifactorial, including (a) an increase in hydrostatic pressure due to large-volume infusions, multiple transfusions, or parenteral nutrition; (b) an increase in pulmonary vessel permeability; (c) the cardiotoxic effects of chemotherapy; and (d) kidney injury [34].

**Figure 10:**
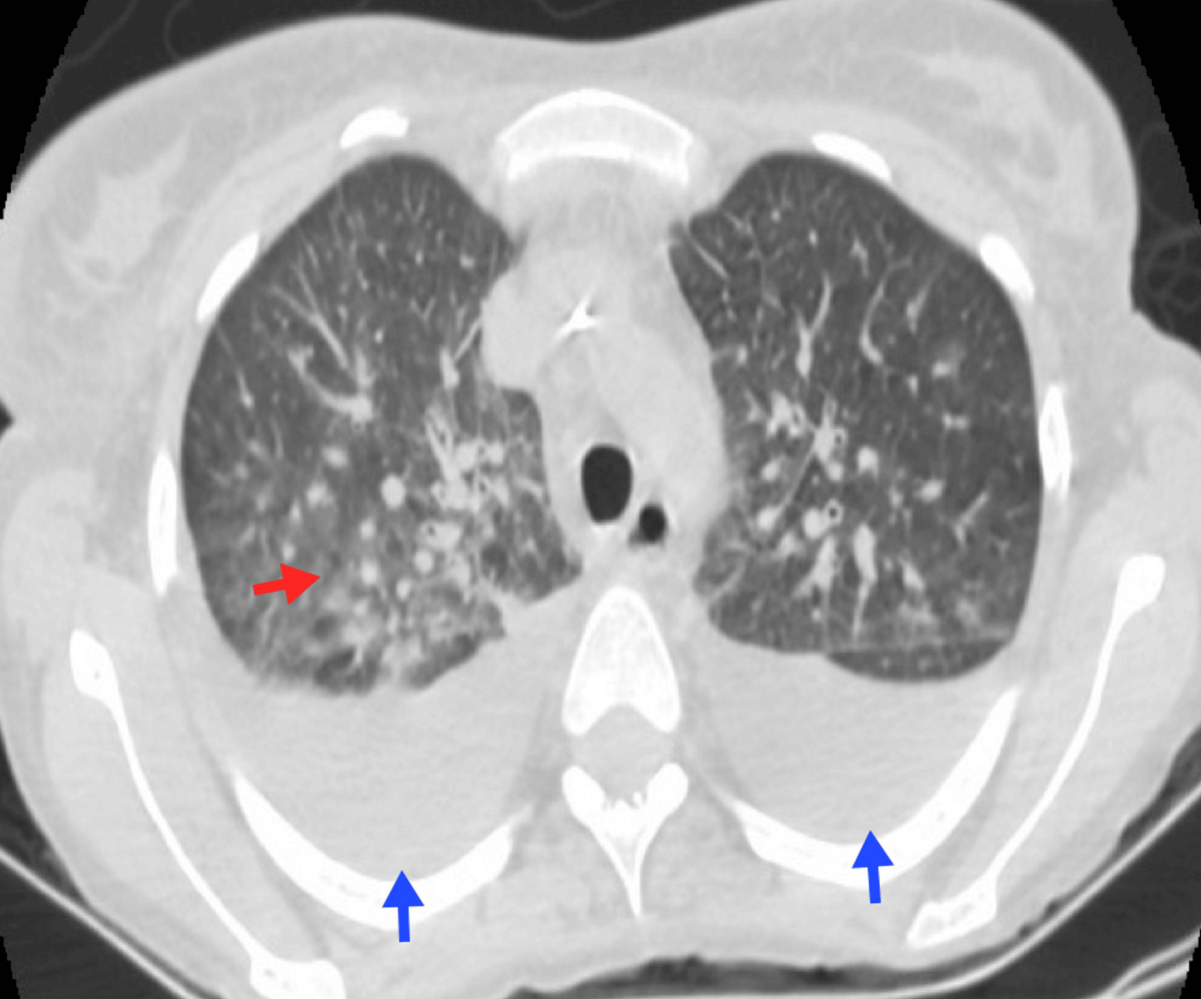
Pulmonary edema A 42-year-old female patient with a blastic crisis due to CML. Chest imaging reveals moderate bilateral pleural effusion associated with bilateral posterior-basal passive atelectasis (blue arrows), smooth thickening of the interlobular septa, and symmetrical bilateral ground-glass opacities suggestive of fluid overload (red arrow). CML, chronic myeloid leukemia Image courtesy: Pulmonology Service of the Internal Medicine Department, University of Antioquia. Consent for open access publication was obtained from the patient.

The typical findings on chest X-ray include cardiomegaly, cephalization of pulmonary veins, interstitial congestion, an increase in vascular pedicle width, Kerley B lines, and peribronchial thickening. In severe cases, perihilar shadow, pleural effusion, ground-glass opacities, and consolidation may be observed [43]. HRCT shows similar findings (Figure 10) [3].

Pulmonary Hypertension

Patients with MPNs and CML are at risk of developing pulmonary hypertension, although the exact prevalence remains unclear, with data primarily limited to case reports and small case series [42]. The underlying pathophysiology is not fully understood, but key mechanisms thought to contribute include chronic thromboembolic pulmonary hypertension (Figure 9), portal hypertension, EMH, chemotherapy toxicity, and invasion of pulmonary vessels [44].

Additionally, certain therapeutic interventions have been linked to pulmonary hypertension. Notably, the second-generation tyrosine kinase inhibitor (TKI) dasatinib, used in the treatment of CML and Philadelphia-positive ALL, has occasionally been associated with severe pre-capillary pulmonary hypertension. The median time from the initiation of dasatinib to the diagnosis of pulmonary hypertension is approximately 34 months (range: eight to 48 months), and the disease progression is typically slow [45].

Pulmonary Veno-Occlusive Disease (PVOD)

PVOD is a syndrome characterized by increased pulmonary vascular resistance. It is a rare cause of symptoms such as fatigue, dizziness, weakness, and dyspnea, particularly after HSCT. Due to its similarity in presentation to pulmonary arterial hypertension, PVOD may be misdiagnosed, and some recent reports suggest that both conditions could be part of the same spectrum [46].

It is hypothesized that PVOD may develop as a result of vascular damage induced by HSCT. The condition involves interstitial pulmonary edema and capillary congestion, caused by fibrous occlusion of postcapillary venules and some larger vessels [47,48].

The typical triad includes increased pulmonary resistance, normal left-sided filling pressures, and pulmonary edema, although the presentation can vary. Treatment generally involves oxygen therapy for hypoxemic patients. In some cases, immunosuppressants and/or pulmonary vasodilators may be used, although their efficacy is variable [49,50].

Pulmonary Alveolar Proteinosis (PAP)

In PAP, surfactant and other amorphous, periodic acid-Schiff (PAS)-positive substances accumulate in the terminal bronchioles and alveoli due to macrophage dysfunction. It has traditionally been classified into three types: congenital, autoimmune, and secondary. Secondary PAP has been linked to MDS, CML, and HSCT, likely due to macrophage depletion or dysfunction or the production of anti-granulocyte-macrophage colony-stimulating factor alloantibodies. However, the role of these antibodies remains controversial [18,51].

Symptoms include cough, dyspnea, hypoxemia, and diffuse alveolar opacities, primarily in the central regions and pulmonary bases [52]. Diagnosis is suggested when a milky BAL fluid is observed, with PAS-positive material found in the macrophages’ cytoplasm, after ruling out infectious causes. Definitive diagnosis typically requires a biopsy [53].

Organizing Pneumonia

**Figure 11:**
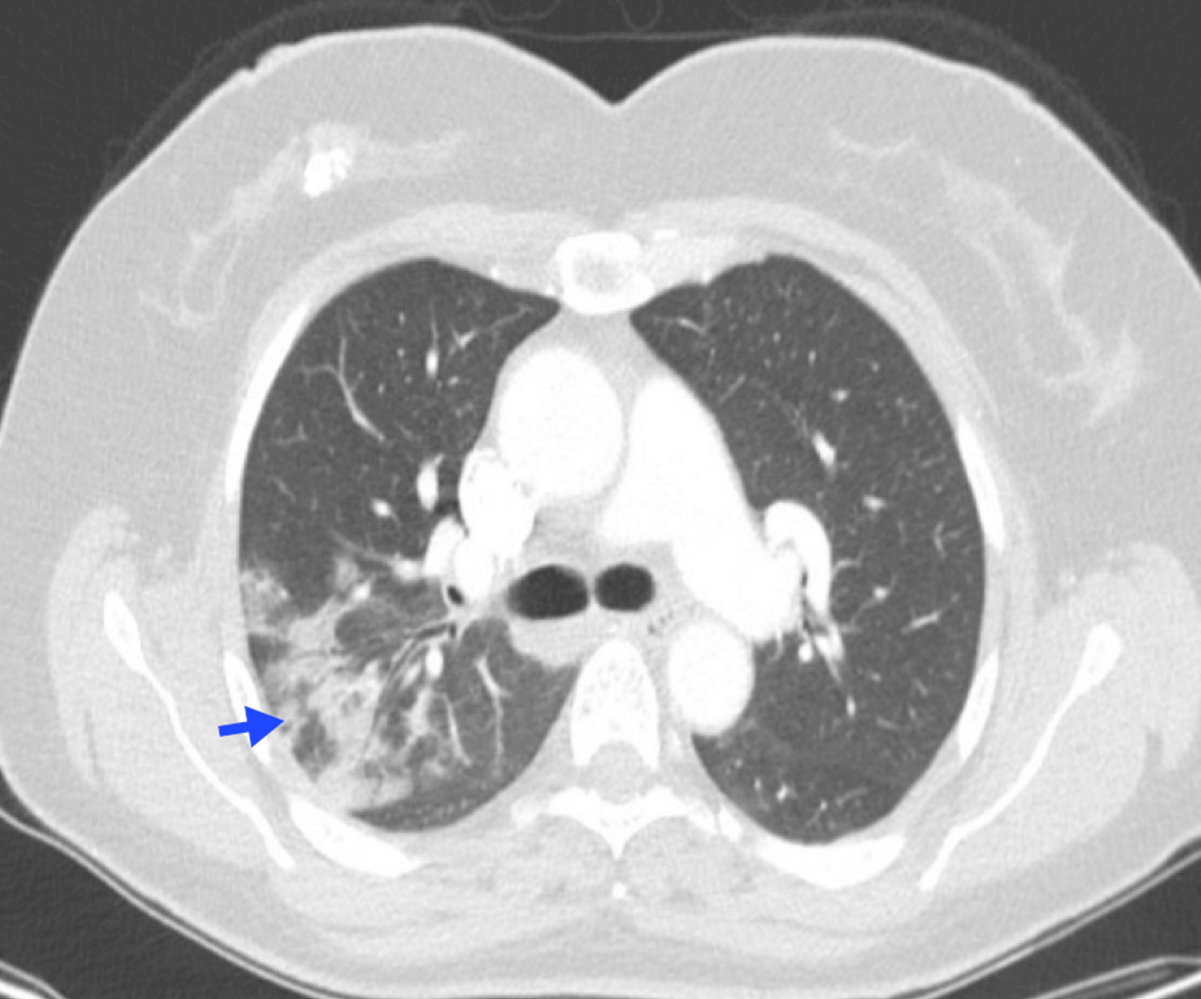
Cryptogenic organizing pneumonia A 53-year-old female patient with splenic marginal zone lymphoma. Chest CT reveals subpleural and peribronchial consolidations, along with multifocal ground-glass opacities in the posterior segment of the upper right lobe and lateral segment of the middle lobe (blue arrow). Image courtesy: Pulmonology Service of the Internal Medicine Department, University of Antioquia. Consent for open access publication was obtained from the patient.

Organizing pneumonia is a rare occurrence in patients with HMs, with only a few exceptional cases reported - 11 out of 17,808 patients [54]. It has been associated with MDS and, more rarely, with myeloproliferative syndromes. This condition is characterized by nonspecific parenchymal inflammation, resulting from the infiltration of inflammatory cells, collagen, and fibroblasts [18]. In imaging studies, low-density alveolar opacities are typically observed (Figure 11).

These lesions are generally migratory, recurrent, and highly responsive to steroid therapy, although diffuse interstitial infiltration and localized pneumonia have also been reported [54].

Chylothorax

**Figure 12:**
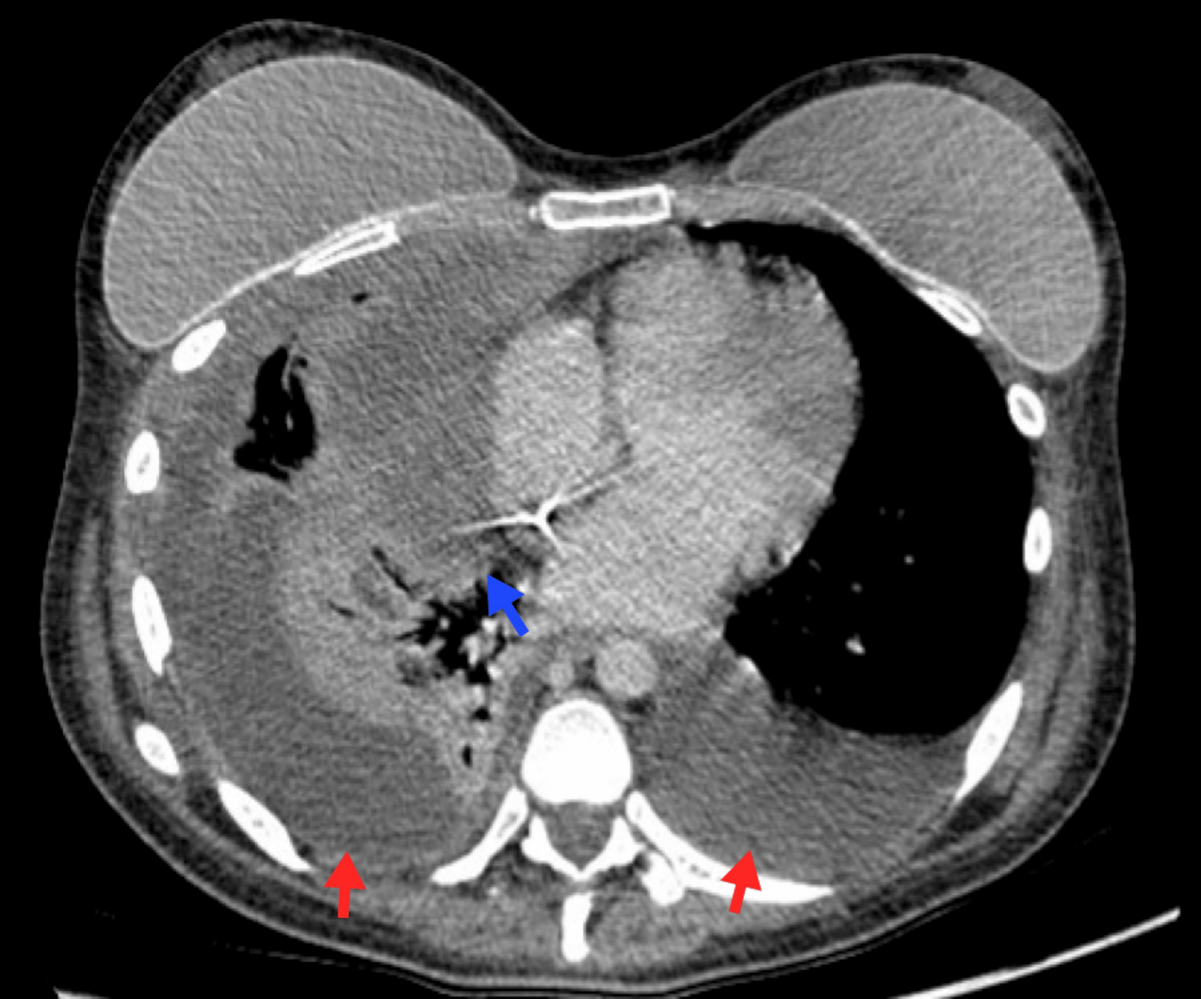
Chylothorax A 26-year-old female patient with primary mediastinal large B-cell lymphoma. Chest imaging reveals a mass in the anterior mediastinum (blue arrow) and moderate bilateral pleural effusion (red arrows). Pleural fluid studies confirm the presence of chylothorax. Image courtesy: Pulmonology Service of the Internal Medicine Department, University of Antioquia. Consent for open access publication was obtained from the patient.

Lymphoproliferative syndromes, primarily NHL, CLL, and Waldenström macroglobulinemia, account for 11% of the etiology of malignant chylothorax (Figure 12) [55].

If the rupture of the thoracic duct occurs below T5-T6, chylothorax typically presents on the right side. However, if the rupture occurs higher, it usually presents on the left side [56]. Most cases are unilateral, with a predominance of right-sided involvement. Chylothorax is often associated with an insidious pleural effusion, accompanied by symptoms such as dyspnea, cough, and weight loss. Pleuritic chest pain and chyloptysis are rare.

The pleural fluid appears milky and is predominantly exudative (86%), with lymphocytic pleocytosis, elevated triglyceride levels (with a diagnostic cutoff of >110 mg/dL), or chylomicrons detected through lipoprotein electrophoresis. Cholesterol levels are typically low (<200 mg/dL) [57-59]. The patient’s medical history, along with initial imaging tests (chest X-ray and CT), often helps identify the underlying cause [60,61].

Complications Due to TKI Use

TKIs are the primary treatment for patients with CML. Imatinib remains the first-line therapy during the chronic phase of the disease, although other second-generation TKIs have been FDA-approved [62]. TKI therapy is associated with various pulmonary complications, including interstitial lung disease (ILD), pleural effusion, and precapillary pulmonary hypertension. Dasatinib is the TKI most strongly linked to pulmonary complications, with pleural effusion occurring in 10-35% of patients [63].

Quintás-Cardama et al. reported a pleural effusion frequency of 35% (48/138) in patients with CML treated with dasatinib. Among these, 10 required thoracentesis and pleural fluid analysis, with 78% of cases being exudative and 22% transudative. The median time from dasatinib initiation to pleural effusion diagnosis was 42 weeks (range: four to 120 weeks). Most patients experienced gradual onset, and some cases were associated with pericardial effusion [64].

ILD is the earliest pulmonary complication associated with TKIs, although the exact frequency is unknown. Ohnishi et al. reported a series of 27 cases from Japan, with the mean time from therapy initiation to symptom development being 49 days (range: 10-282 days) and the median dose 400 mg (range: 200-600 mg). No significant association was found between the dose and the occurrence of ILD [65].

**Figure 13:**
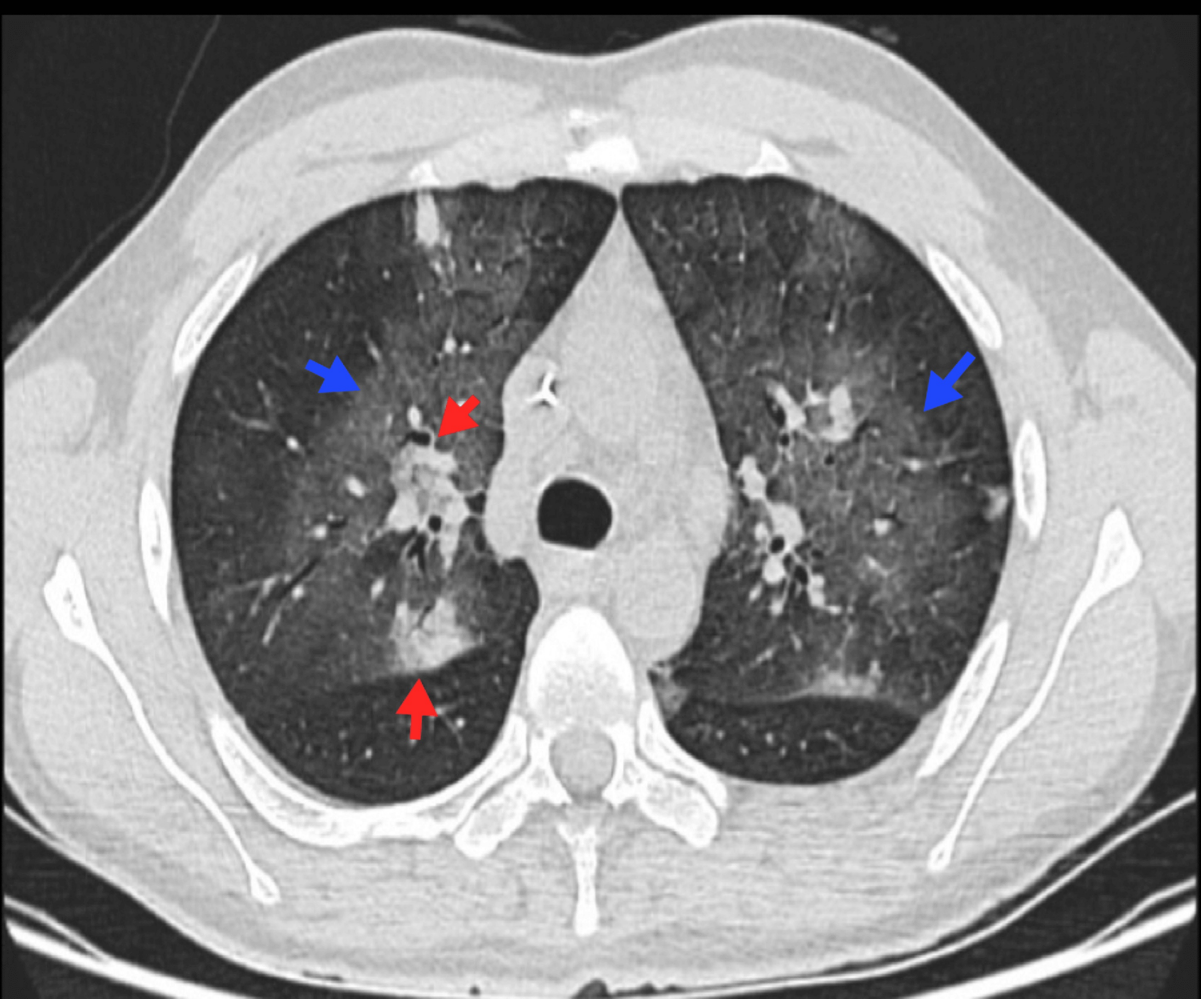
Differentiation syndrome and opportunistic mycosis A 22-year-old male patient with acute promyelocytic leukemia developed dyspnea after 10 days of ATRA therapy. Chest CT revealed central ground-glass opacities in the lung parenchyma, predominantly in the upper and middle lobes (blue arrows), raising suspicion for differentiation syndrome. In addition, pulmonary nodules and peribronchovascular consolidations were observed (red arrows), and galactomannan antigen was positive in the BAL. Treatment for suspected aspergillosis with voriconazole was initiated, resulting in clinical improvement. ATRA, all-trans retinoic acid; BAL, bronchoalveolar lavage Image courtesy: Pulmonology Service of the Internal Medicine Department, University of Antioquia. Consent for open access publication was obtained from the patient.

The mechanisms behind TKI-induced ILD remain unclear. Histological analysis shows both cytotoxic and noncytotoxic origins, with findings ranging from bronchiolitis obliterans organizing pneumonia to DAD [65]. Clinically, symptoms range from cough, dyspnea, fever, and hypoxemia to sudden respiratory failure, with the disease course varying widely. Chest X-ray findings often show bilateral, basal infiltrates, although they can be patchy and migratory. HRCT may reveal ground-glass opacities, consolidations, reticular opacities, or a combination of these findings (Figure 13) [63].

Complications Due to ICIs

ICIs are a new class of drugs offering an innovative approach to treating various malignancies, especially solid tumors. Among HMs, HL has shown the greatest clinical benefits. ICIs also seem to have a synergistic role in augmenting clinical responses in HSCT [66]. However, the administration of ICIs can lead to immune-related adverse events (irAEs), with ILD being a rare but potentially severe complication.

In a retrospective study of patients primarily receiving programmed cell death-1 inhibitors, 64 out of 1826 cancer patients (3.5%) developed ICI-related ILD. This complication primarily occurred in males, often those who were former or current smokers, with a median age of 59 years. Severity levels included grade 2/3 in 65.6%, grade 4 in 9.4%, and fatal cases in 9.4%. CT scans predominantly showed ground-glass opacities (81.3%), followed by consolidations (53.1%). The most common histologic patterns were organizing pneumonia (23.4%) and hypersensitivity pneumonitis (15.6%) [67]. However, these findings are mainly based on patients with lung cancer or melanoma, and data on HM patients are limited [66]. Despite ICI-induced ILD being one of the more common serious irAEs, treatment options remain limited, partly due to a lack of understanding of the mechanisms behind its development [67].

Infectious pulmonary complications

Specific immune defects can predispose individuals to a wide range of infections. Neutropenia, for example, increases susceptibility to infections caused by Gram-negative bacilli, Gram-positive cocci, and fungi. T-cell dysfunction, in contrast, heightens the risk of intracellular bacterial infections, viral infections, fungal infections, and parasitic infections. Meanwhile, dysfunction in B cells and humoral immunity makes individuals more vulnerable to infections caused by encapsulated bacteria and *Mycoplasma* spp. (Table 2, Table 3) [68].

**Table 2:** Immune disorders and related pathogens in HMs and HSCT ^*^ Neutropenia is the most common individual risk factor for cancer-associated pneumonia [66]. ^**^ Pulmonary complications during the pre-transplant stage are associated with lower mortality compared to those that develop in the late stage [66]. HM, hematologic malignancy; HSCT, hematopoietic stem cell transplantation; MDS, myelodysplastic syndrome; MM, multiple myeloma

Immune disorder	Potential causes	Related pathogens
Neutrophile function/number^*^	Leukemias, lymphomas, MDSs, cytoreductive therapy, and corticosteroids	Gram-negative bacilli, Gram-positive cocci, and fungi (*Aspergillus *spp., Mucorales, *Fusarium *spp., and *Scedosporium *spp.)
	HSCT and its stages: (a) pre-transplant stage: first 30 days; (b) post-transplant stage: early: 30-100 days due to cellular immunity disorders and late: >100 days	Pre-transplant stage: higher risk of Gram-negative infection^**^
T lymphocytes	Lymphomas, corticosteroids, T cell depletion, and drugs	Intracellular bacteria (*Nocardia*, Mycobacterium, and *Legionella*), viruses (respiratory viruses and Herpesviridae). Fungi (*Pneumocystis jirovecii*, *Cryptococcus*spp., *Histoplasma capsulatum*, *Coccidioides *spp., *Aspergillus *spp., Mucorales, *Fusarium*, and *Scedosporium*), and parasites (*Strongyloides *spp. and *Toxoplasma *spp.)
B lymphocytes and humoral immunity	Leukemias, MM, anti-B-Cell antibodies, splenectomy, plasmapheresis, and drugs	Encapsulated bacteria (Pneumococcus and *Haemophilus influenzae*) and *Mycoplasma *spp.

**Table 3:** Major infectious manifestations in patients with HMs ^* ^The most characteristic radiologic patterns of infectious complications in the lungs [66]. BAL, bronchoalveolar lavage; HM, hematologic malignancy

Infectious agent	Main agents	CT findings	Definitive diagnosis
Bacterial	*Pseudomonas* spp., *Nocardia*, *Legionella*, *Haemophilus influenzae*, *Enterobacter*, and *Mycobacterium tuberculosis*	Segmentary or lobar consolidation; centrilobular tree in bud pattern^*^	BAL and fibrobronchoscopy
Fungal	*Aspergillus* (*Aspergillus flavus*), Mucorales (*Rhizopus*), *Fusarium *spp., and *Scedosporium *spp.	Halo sign (angioinvasive aspergillosis or mucormycosis), variable size nodules (0.5-5 cm diameter) (angioinvasive aspergillosis), reverse halo sign (Mucorales infection), nodules, consolidation and cavitation (disseminated candidiasis), ground-glass (*Pneumocystis jirovecii*infection), and lobar consolidation (differential diagnosis with bacterial pneumonia)^*^	BAL and fibrobronchoscopy with specific markers for each microorganism/open lung biopsy
Viral	Cytomegalovirus, rhinovirus, adenovirus, influenza, parainfluenza, syncytial respiratory virus, metapneumovirus, and SARS-CoV-2	Ground-glass, consolidations, centrilobular nodules, and reticular opacities^*^	BAL and fibrobronchoscopy with PCR detection

**Figure 14:**
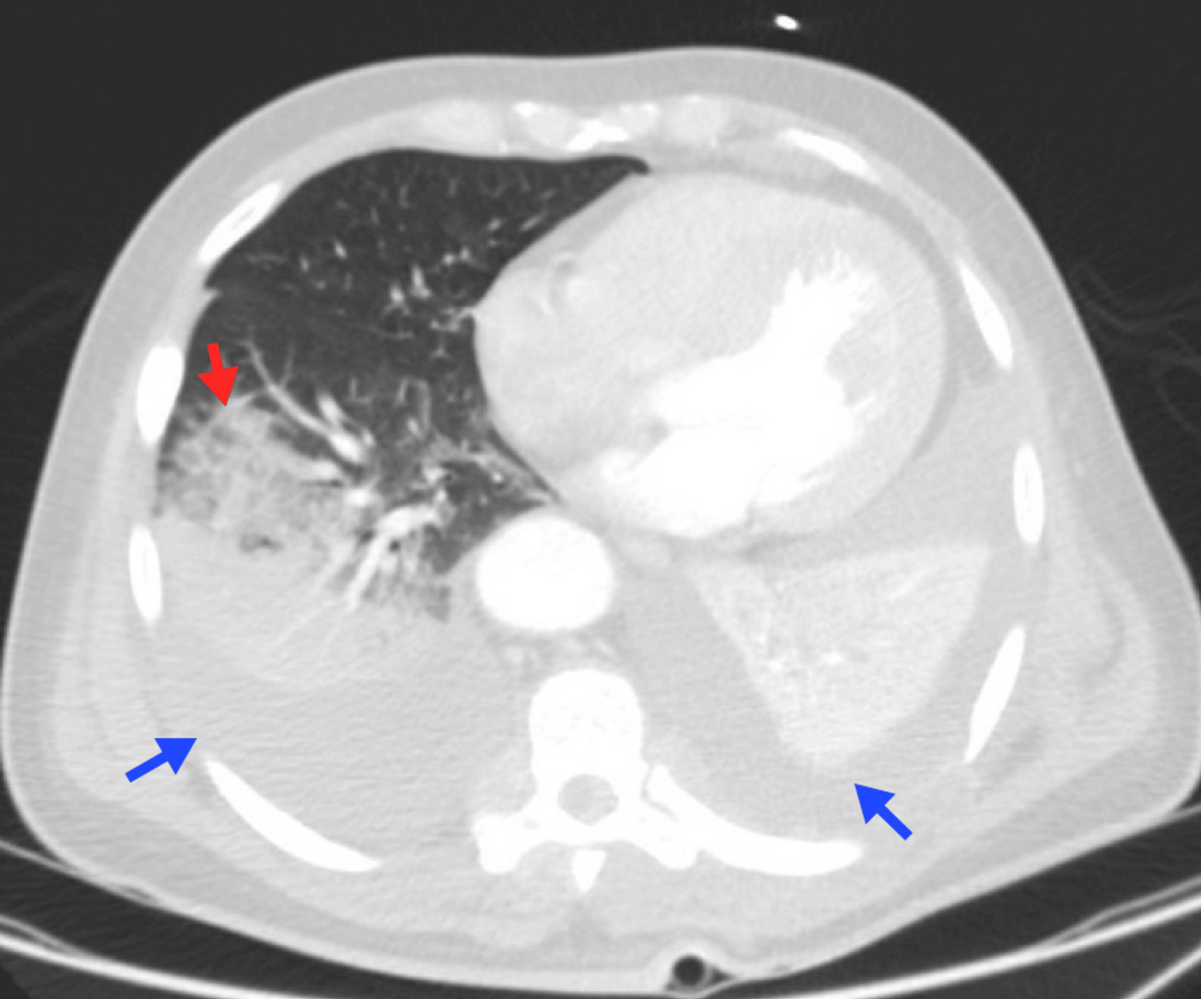
Bacterial pneumonia A 59-year-old male with lambda MM. Chest CT reveals extensive bilateral pleural effusion (blue arrows), along with alveolar consolidation in the inferior right lobe due to pneumonic involvement (red arrow). *Haemophilus influenzae* was isolated in microbiological studies. MM, multiple myeloma Image courtesy: Pulmonology Service of the Internal Medicine Department, University of Antioquia. Consent for open access publication was obtained from the patient.

**Figure 15:**
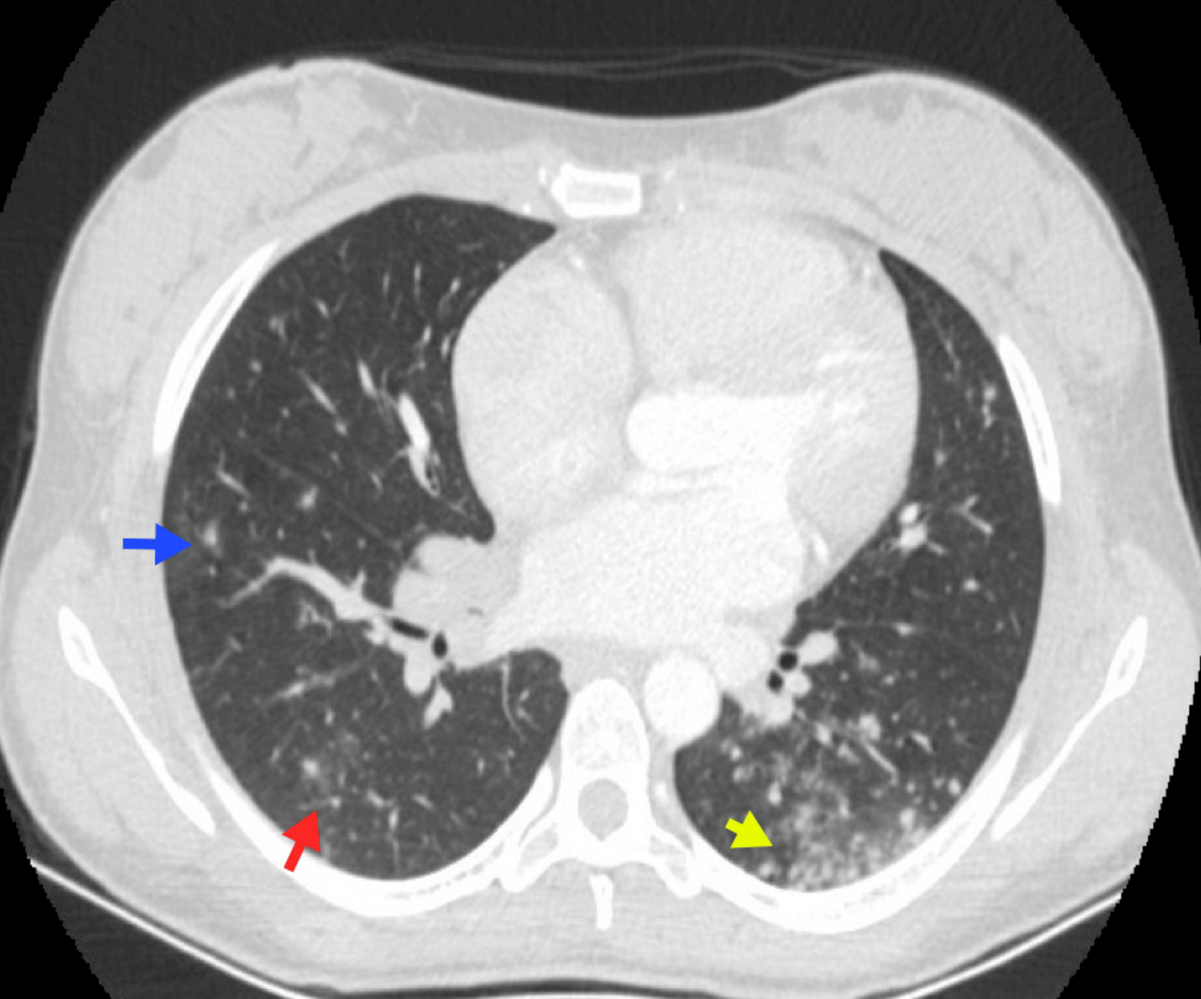
Fungal infection A 28-year-old female patient with nongerminal center diffuse large B-cell lymphoma and a prior history of cryptococcal meningitis. Chest CT reveals multiple centrilobular nodules in the lung parenchyma (blue arrow), ground-glass opacities in both lungs (red arrow), and basal consolidation in the left lung (yellow arrow). *Cryptococcus* spp. was isolated from the BAL. BAL, bronchoalveolar lavage Image courtesy: Pulmonology Service of the Internal Medicine Department, University of Antioquia. Consent for open access publication was obtained from the patient.

Figure 13, Figure 14, and Figure 15 illustrate examples of pulmonary infections in patients with HMs.

Research agenda

More precise data are needed regarding the prevalence of pulmonary manifestations associated with the toxicity of ICIs in the context of HM treatments. Further research into the pathophysiology of complications such as pulmonary hypertension in MPNs or TKI-induced ILD is essential to develop more targeted therapeutic approaches.

## Conclusions

Pulmonary complications are common in patients with HMs and can result from both infectious and noninfectious causes. The underlying hematologic condition, treatment regimens, and individual risk factors should guide the diagnostic approach. Radiographic imaging, particularly HRCT, plays a crucial role in evaluating pulmonary manifestations and can help differentiate between various patterns of involvement. It is important to consider antineoplastic therapies, such as chemotherapy, TKIs, ICIs, and HSCT, as significant contributors to pulmonary toxicity. A multidisciplinary approach involving hematologists, pulmonologists, radiologists, and other specialists is vital for ensuring accurate diagnosis and appropriate management of these complications.

## References

[1] Cheng GS, Possick JD (2017). Pulmonary disease in non-pulmonary malignancy. Clin Chest Med.

[2] Kocarnik JM, Compton K, Dean FE (2022). Cancer incidence, mortality, years of life lost, years lived with disability, and disability-adjusted life years for 29 cancer groups from 2010 to 2019: a systematic analysis for the Global Burden of Disease Study 2019. JAMA Oncol.

[3] Choi MH, Jung JI, Chung WD (2014). Acute pulmonary complications in patients with hematologic malignancies. Radiographics.

[4] Harris B, Morjaria SM, Littmann ER (2016). Gut microbiota predict pulmonary infiltrates after allogeneic hematopoietic cell transplantation. Am J Respir Crit Care Med.

[5] Angirish B, Sanghavi P, Jankharia B (2020). Pulmonary manifestations of lymphoma: a pictorial essay. Lung India.

[6] Hare SS, Souza CA, Bain G, Seely JM, Frcpc Frcpc, Gomes MM, Quigley M (2012). The radiological spectrum of pulmonary lymphoproliferative disease. Br J Radiol.

[7] Diederich S, Link TM, Zühlsdorf H, Steinmeyer E, Wormanns D, Heindel W (2001). Pulmonary manifestations of Hodgkin's disease: radiographic and CT findings. Eur Radiol.

[8] Bosanko CM, Korobkin M, Fantone JC, Rubin SB, Lynch JP (1991). Lobar primary pulmonary lymphoma: CT findings. J Comput Assist Tomogr.

[9] Austin JH, Müller NL, Friedman PJ (1996). Glossary of terms for CT of the lungs: recommendations of the Nomenclature Committee of the Fleischner Society. Radiology.

[10] Ahmed S, Siddiqui AK, Rossoff L, Sison CP, Rai KR (2003). Pulmonary complications in chronic lymphocytic leukemia. Cancer.

[11] Berkman N, Polliack A, Breuer R, Okon E, Kramer M (1992). Pulmonary involvement as the major manifestation of chronic lymphocytic leukemia. Leuk Lymphoma.

[12] Faiz SA, Sahay S, Jimenez CA (2014). Pleural effusions in acute and chronic leukemia and myelodysplastic syndrome. Curr Opin Pulm Med.

[13] Ross JS, Ellman L (1974). Leukemic infiltration of the lungs in the chemotherapeutic era. Am J Clin Pathol.

[14] Wang HQ, Li J (2016). Clinicopathological features of myeloid sarcoma: report of 39 cases and literature review. Pathol Res Pract.

[15] Paydas S, Zorludemir S, Ergin M (2006). Granulocytic sarcoma: 32 cases and review of the literature. Leuk Lymphoma.

[16] Bashoura L, Eapen GA, Faiz SA (2017). Pulmonary manifestations of lymphoma and leukemia. Clin Chest Med.

[17] Koch CA, Li CY, Mesa RA, Tefferi A (2003). Nonhepatosplenic extramedullary hematopoiesis: associated diseases, pathology, clinical course, and treatment. Mayo Clin Proc.

[18] Lamour C, Bergeron A (2011). Non-infectious pulmonary complications of myelodysplastic syndromes and chronic myeloproliferative disorders. Rev Mal Respir.

[19] Guizetti P (1912). Haemolyticlier korpentaler icterus. Buts Z Pathol Anat UZ Allg Pathol.

[20] Elbers H, vd Stadt J, Wagenaar SS (1980). Tumor-simulating thoracic extramedullary hematopoiesis. Ann Thorac Surg.

[21] Neiman RS, Barcos M, Berard C (1981). Granulocytic sarcoma: a clinicopathologic study of 61 biopsied cases. Cancer.

[22] Takasugi JE, Godwin JD, Marglin SI, Petersdorf SH (1996). Intrathoracic granulocytic sarcomas. J Thorac Imaging.

[23] Enright H, Jacob HS, Vercellotti G, Howe R, Belzer M, Miller W (1995). Paraneoplastic autoimmune phenomena in patients with myelodysplastic syndromes: response to immunosuppressive therapy. Br J Haematol.

[24] Cohen PR, Talpaz M, Kurzrock R (1988). Malignancy-associated Sweet's syndrome: review of the world literature. J Clin Oncol.

[25] Niţu M, CriȘan E, Olteanu M, Călăraşu C, Olteanu M, Popescu MR (2014). Lung involvement in multiple myeloma - case study. Curr Health Sci J.

[26] Zhou Y, Wang XH, Meng SS, Wang HC, Li YX, Xu R, Lin XH (2020). Primary pulmonary plasmacytoma accompanied by overlap syndrome: a case report and review of the literature. World J Clin Cases.

[27] Cao W, Liang S, Liu J, Bai J, Li H (2015). Castleman's disease presenting in the lungs: a report of two cases. Oncol Lett.

[28] Vieira IG, Marchiori E, Zanetti G, Cabral RF, Takayassu TC, Spilberg G, Batista RR (2009). Pulmonary amyloidosis with calcified nodules and masses - a six-year computed tomography follow-up: a case report. Cases J.

[29] Slanetz PJ, Whitman GJ, Shepard JA, Chew FS (1994). Nodular pulmonary amyloidosis. AJR Am J Roentgenol.

[30] Tenholder MF, Hooper RG (1980). Pulmonary infiltrates in leukemia. Chest.

[31] Worthy SA, Flint JD, Müller NL (1997). Pulmonary complications after bone marrow transplantation: high-resolution CT and pathologic findings. Radiographics.

[32] Witte RJ, Gurney JW, Robbins RA (1991). Diffuse pulmonary alveolar hemorrhage after bone marrow transplantation: radiographic findings in 39 patients. AJR Am J Roentgenol.

[33] Primack SL, Miller RR, Müller NL (1995). Diffuse pulmonary hemorrhage: clinical, pathologic, and imaging features. AJR Am J Roentgenol.

[34] Soubani AO, Miller KB, Hassoun PM (1996). Pulmonary complications of bone marrow transplantation. Chest.

[35] Tanaka N, Matsumoto T, Miura G, Emoto T, Matsunaga N (2002). HRCT findings of chest complications in patients with leukemia. Eur Radiol.

[36] van Buchem MA, Wondergem JH, Kool LJ, te Velde J, Kluin PM, Bode PJ, Busscher DL (1987). Pulmonary leukostasis: radiologic-pathologic study. Radiology.

[37] Tanaka N, Matsumoto T, Miura G, Emoto T, Matsunaga N, Satoh Y, Oka Y (2002). CT findings of leukemic pulmonary infiltration with pathologic correlation. Eur Radiol.

[38] Blom JW, Doggen CJ, Osanto S, Rosendaal FR (2005). Malignancies, prothrombotic mutations, and the risk of venous thrombosis. JAMA.

[39] Murchison JT, Wylie L, Stockton DL (2004). Excess risk of cancer in patients with primary venous thromboembolism: a national, population-based cohort study. Br J Cancer.

[40] Vu K, Luong NV, Hubbard J (2015). A retrospective study of venous thromboembolism in acute leukemia patients treated at the University of Texas MD Anderson Cancer Center. Cancer Med.

[41] Falanga A, Marchetti M (2012). Thrombotic disease in the myeloproliferative neoplasms. Hematology Am Soc Hematol Educ Program.

[42] Guilpain P, Montani D, Damaj G (2008). Pulmonary hypertension associated with myeloproliferative disorders: a retrospective study of ten cases. Respiration.

[43] Benya EC, Goldman S (1997). Bone marrow transplantation in children: imaging assessment of complications. Pediatr Clin North Am.

[44] Machado RF, Farber HW (2013). Pulmonary hypertension associated with chronic hemolytic anemia and other blood disorders. Clin Chest Med.

[45] Montani D, Bergot E, Günther S (2012). Pulmonary arterial hypertension in patients treated by dasatinib. Circulation.

[46] Simonneau G, Gatzoulis MA, Adatia I (2013). Updated clinical classification of pulmonary hypertension. J Am Coll Cardiol.

[47] Jodele S, Hirsch R, Laskin B, Davies S, Witte D, Chima R (2013). Pulmonary arterial hypertension in pediatric patients with hematopoietic stem cell transplant-associated thrombotic microangiopathy. Biol Blood Marrow Transplant.

[48] Troussard X, Bernaudin JF, Cordonnier C, Fleury J, Payen D, Briere J, Vernant JP (1984). Pulmonary veno-occlusive disease after bone marrow transplantation. Thorax.

[49] Rambihar VS, Fallen EL, Cairns JA (1979). Pulmonary veno-occlusive disease: antemortem diagnosis from roentgenographic and hemodynamic findings. Can Med Assoc J.

[50] Vande Vusse LK, Madtes DK (2017). Early onset noninfectious pulmonary syndromes after hematopoietic cell transplantation. Clin Chest Med.

[51] Sanderson JE, Spiro SG, Hendry AT, Turner-Warwick M (1977). A case of pulmonary veno-occlusive disease respondong to treatment with azathioprine. Thorax.

[52] Kuroda T, Hirota H, Masaki M (2006). Sildenafil as adjunct therapy to high-dose epoprostenol in a patient with pulmonary veno-occlusive disease. Heart Lung Circ.

[53] Rosen SH, Castleman B, Liebow AA, Enzinger FM, Hunt RT (1958). Pulmonary alveolar proteinosis. N Engl J Med.

[54] Trapnell BC, Whitsett JA, Nakata K (2003). Pulmonary alveolar proteinosis. N Engl J Med.

[55] Krūmiņa A, Auziņa D, Legzdiņa A, Lejniece S (2023). Chylothorax: a tangled road to definitive diagnosis of non-Hodgkin lymphoma. Am J Case Rep.

[56] Fukumoto A, Terao T, Kuzume A (2022). Management of lymphoma-associated chylothorax by interventional radiology and chemotherapy: a report of five cases. Int J Hematol.

[57] Pospiskova J, Smolej L, Belada D (2019). Experiences in the treatment of refractory chylothorax associated with lymphoproliferative disorders. Orphanet J Rare Dis.

[58] Cordier JF (2000). Organising pneumonia. Thorax.

[59] Doerr CH, Allen MS, Nichols FC 3rd, Ryu JH (2005). Etiology of chylothorax in 203 patients. Mayo Clin Proc.

[60] Sahn SA (2006). Pleural effusions of extravascular origin. Clin Chest Med.

[61] Maldonado F, Hawkins FJ, Daniels CE, Doerr CH, Decker PA, Ryu JH (2009). Pleural fluid characteristics of chylothorax. Mayo Clin Proc.

[62] Cohen P, Cross D, Jänne PA (2021). Kinase drug discovery 20 years after imatinib: progress and future directions. Nat Rev Drug Discov.

[63] Barber NA, Ganti AK (2011). Pulmonary toxicities from targeted therapies: a review. Target Oncol.

[64] Quintás-Cardama A, Kantarjian H, O'brien S, Borthakur G, Bruzzi J, Munden R, Cortes J (2007). Pleural effusion in patients with chronic myelogenous leukemia treated with dasatinib after imatinib failure. J Clin Oncol.

[65] Ohnishi K, Sakai F, Kudoh S, Ohno R (2006). Twenty-seven cases of drug-induced interstitial lung disease associated with imatinib mesylate. Leukemia.

[66] Tsumura A, Levis D, Tuscano JM (2023). Checkpoint inhibition in hematologic malignancies. Front Oncol.

[67] Delaunay M, Cadranel J, Lusque A (2017). Immune-checkpoint inhibitors associated with interstitial lung disease in cancer patients. Eur Respir J.

[68] Bommart S, Bourdin A, Makinson A (2013). Infectious chest complications in haematological malignancies. Diagn Interv Imaging.

